# An experimental investigation of laser scabbling on cement-based materials using nanosecond fiber laser

**DOI:** 10.1038/s41598-022-16301-4

**Published:** 2022-07-16

**Authors:** Tam Van Huynh, Mounarik Mondal, Dongkyoung Lee

**Affiliations:** 1 Department of Future Convergence Engineering, Kongju National University, Cheonan, 31080 Korea; 2 Department of Mechanical and Automotive Engineering, Kongju National University, Cheonan, 31080 Korea; 3 Center for Advanced Powder Materials and Parts of Powder (CAMP2), Kongju National University, Cheonan, 31080 South Korea

**Keywords:** Energy science and technology, Engineering, Materials science, Physics

## Abstract

In this study, the influence of a pulsed fiber laser of 250 W power with a spot size of 40 µm was successfully analyzed during scabbling of six types of cement mortar and three types of ultra-high-performance concrete (UHPC). Confocal microscopy on the surface of the scabbled samples elucidated the formation of three distinct zones: glassy layer (GL), partially melted zone (PMZ), and heat-affected zone (HAZ) with unique morphological appearances. The glassy layer exhibited bubble formation, whereas cracks were spotted alongside the scabbled area. The difference in scabbling depth between the beginning and end of the process was revealed by using 3D topography images. Moreover, the development of pores and the changes in the microstructure of each zone were observed by using scanning electron microscopy (SEM). Further energy dispersive X-ray (EDX) analysis also revealed significant changes in the percentage of silicon and calcium inside the glassy layer and non-processed zone (NPZ).

## Introduction

Cement-based materials are widely used in many construction sites due to their low cost, availability, engineering properties, and durability. They are used for constructing civil and industrial structures like factory production units and power plant. In current time, power plants are extremely important for the growth of any civic society. Over the past few years, fossil fuel-driven power plants were gradually transformed into nuclear-fueled power plants intending to decrease fossil fuel consumption and CO_2_ emission^1,2^. However, with the emergence of green energy and to deal with safety aspects in the modern era, more than 170 nuclear reactors have been shut down permanently in Korea for the last four years. In the previous 40 years, more than 85 commercial power reactors, 45 experimental or prototype power reactors, over 250 research reactors, and many fuel cycle facilities have retired from regular operation^3,4^.

Consequently, this has enforced an increased number of decommissioning and decontamination of nuclear power plant concrete structures and components. It is specifically reported that 750 and 900 tons of radioactive concrete materials can be generated in the dismantling of gas-cooled and pressurized water reactors. Dismantling of the Korean research reactor (KKR-2) produced 260 tons of radioactive concrete and more than 60 tons of uranium compound contaminated concrete wastes^5,6^. The generation of this radioactive concrete is mainly attributed to the exposure of radioactive liquids and aerosols during the operation. Furthermore, its thickness percentage grows higher inside the structures near the nuclear reactors. Disposal of this waste product can be relatively costly and challenging as it has to adhere to the rules of nuclear waste disposal^7^.

Thus, to decrease the volume of nuclear waste during decommissioning after prolonged usage of nearly 40 years, 8–12 mm thick of contaminated concrete layer is removed by either mechanical, chemical, or biological process^5,8^. The traditional destructive mechanical process like shaving, abrasive blasting, and scabbling mainly results in fine secondary waste products, rough finishing, high vibration, involvement of heavy equipment, and consumption of higher labor costs. Specifically, the production of fine secondary waste products sometimes also poses the threat of causing anoxia to the nearby workers^9^. Furthermore, in the case of the chemical and biological non-destructive process, secondary waste generation is reduced. However, the secondary chemical waste in the form of liquid or slurry contaminates the groundwater or flows to the ocean, hampering the natural water habitat. In addition, for the chemical decontamination, harmful acids and chelating agents are used, which can also cause harm to the operators due to toxic fumes. Apart from natural hazards, the depth of material removed by the chemical and biological decontaminations is limited and can be highly time-consuming^5^.

On the other hand, laser processing has several advantages: remote control, non-contact process, high precision, no-requirement of secondary media assisting the process, no-reaction forces, low noise, vibrations, and dust^10^. However, some researchers believe that laser processing can generate some dust particles in a respirable regime, but those problems can be alleviated by attaching dust extraction systems. Due to its rapid development, laser processing is a promising technology to remove defects on concrete surfaces at construction sites. The laser processing capability on concrete has been the subject of various studies^10–13^. Laser cutting has been used in building and construction sites for decades due to its outstanding benefits. In 1994, with an output of 9 kW and a cutting speed of 0.4 cm/min, a CO_2_ laser was demonstrated to cut approximately 300 mm concrete blocks^14^. However, owing to the operational difficulties of the laser system, this technique has not been adopted. The application of fiber laser has provided the operability and flexibility of laser systems to overcome the operation drawbacks. Many studies have been carried out to investigate the laser processing of concrete. Most of the studies have concentrated on laser cutting and drilling of concrete. Nagai et al.^15^ conducted a study to determine the cutting depth on different compressive strength concretes by simultaneously using 6 kW and 9 kW power lasers. The major result showed that the cutting difference between the beginning and ending points depended on the level of the laser heat accumulation. In addition, Lee et al.^16^ also investigated the effect of laser scanning speed on cutting cement-based material. The cutting characteristics according to laser speeds were reported. The effect of adding silica sand into the cement-based material decreased the penetration depth. The surface of the samples after the experiment was also documented. Furthermore, Nguyen et al.^17^ studied the removal of concrete by using a high-power quasicontinuous fiber laser. The experiments were carried out to test the removal performance by upward and downward laser irradiation. The findings pointed out that with the effect of gravity, the upward laser irradiation showed improved performance in concrete removal.

Laser technology was also used in concrete glazing, apart from the extensive range of applications using lasers in the cutting and drilling of concrete. Lawrence et al.^18^ exploited a 2.5 kW high power diode laser (HPDL) to increase the wear resistance of the concrete surface by glazing it. Furthermore, a comparative study of laser glazing between CO_2_ and high-power diode laser (HPDL) was carried out to demonstrate the performance of each laser in glazing concrete. This study analyzed and presented the characteristics of microstructure and morphology of the laser glazed concrete^19^.

As discussed above, laser technology has been significantly advanced and utilized in many fields during the last three decades. However, high-density power lasers in scabbling ordinary concrete in the construction field have not been reported much and require further in-depth analysis to understand its interaction nature with the concrete. Peach et al.^20–23^ investigated the laser scabbling of concrete materials using a low laser power density (laser power density of 176.83 W/cm^2^; laser power of a 5 kW CO_2_ laser and a spot size diameter of 60 mm). The authors reported that the major factors in the mechanisms of this scabbling method were thermal stress and pore pressure spalling. These results were achieved due to the scabbling condition of the low-density power laser. The authors established significant criteria affecting the laser scabbling of concrete. The relationships between laser interaction time, surface temperature, and removal volume were studied in various concrete types. Besides, Heo et al.^24^ also used a laser source of 5 kW with a stand-off distance of 900 mm to carry out the effect of moisture content and mixing proportion of concrete in laser scabbling. The efficiency of laser scabbling for air dry and saturated surface dry concrete is higher than oven dry concrete. The concrete spalling was also confirmed as a major mechanism by this laser scabbling method. Moreover, Huynh et al.^25^ carried out laser scabbling on cement mortars to study the effect of silica sand proportion on scabbling depth and change in color inside the scabbled zone.

The current study investigates the interaction of high-power density laser with concrete of nine different compositions. For each type of concrete composition, three different laser scanning speeds were varied to study their effects on external appearance in color-changing, bubble generation, and crack formation. The present study is investigated by characterizing the generated glassy layer, partially melted zone, heat affected zone, and non-processed zone by analyzing its morphological and elemental composition changes on the surface and cross-sectional region. Further, the effect of laser speed and concrete composition on scabbled depth was also reported. The identification of the glassy layer zone is confirmed by EDX line scanning analysis.

## Experimental procedure

### Materials and mixing procedure

The commercialized cement used in this study was ordinary Portland cement (OPC) type 1, taken from Korea with a mean particle size of 0.35 µm. Silica fume (Elkem 940U; mean particle size 0.30 µm) was used as a binding material to fabricate the UHPC. For the laser scabbling experiment, nine types of cement-based materials, including UHPCs and mortars, were prepared according to the matrix proportions, as shown in Table 1. Besides, two types of silica sand were used, with an average size of 0.25 mm and 1.45 mm. Absolute mass was used to calculate mixed proportions, and the components were mixed using a laboratory mixer. Cement mortar was composed of three main components of cement, silica sand, and water. In this study, the CM and LP labels were used to indicate two different types of cement mortar. In the case of CM, there were three CM types with varying proportions of silica sand in sample mixing to assess the influence of silica sand proportion in the laser scabbling process. Whereas, in the case of LP, there were three-LP types with different water proportions in sample mixing to evaluate the effect of water proportion in the laser scabbling process. The cement mortar samples were prepared by dry-mixing cement and silica sand for 5 min, then water was added, and the mixing was carried out for 3 more minutes. In the case of UHPC samples, the cement and silica sand were also dry-mixed for 5 min first; next, silica fume and silica powder were gently added and dry-mixed for 3 min. After dry-mixing, water was added and further mixed for about 3 min. A polycarboxylate-based superplasticizer with 25% wt solid content by weight was continuously added to ensure the flowability of the fresh mixture of UHPC at a low ratio of water/binder. As per the earlier study, silica fume and silica powder in UHPC mixtures can act as fine aggregates, enhancing hydration and increasing UHPC’s compressive strength higher than 150 MPa, bonding characteristics, rapid generation of hydration products, high ductility, and excellent durability^26–29^. Due to the obvious optimum packing density and limited usage of coarse aggregates, UHPC is defined as more homogeneous than normal strength concrete compared to ordinary cement mortar, as mentioned above. All the mortar and UHPC samples were cast in a prism-shaped mold with the dimensions of 80 × 80 × 15 mm^3^ in the absence of any external vibration. The cast specimens were covered with a plastic sheet and stored at room temperature for 24 h. Then, all specimens were demolded and cured in water for 28 days at 25 °C. Finally, all samples were removed from the water and dried in a laboratory drier at 90 °C for 5 h to remove the water.

**Table 1:** Experimental samples mix design (Proportion by mass).

Series	Cement	Water	Silica sand type 1 (1.45 mm)	Silica sand type 2 (0.25 mm)	Silica fume	Silica powder	Superplasticizer
CM0.2	1	0.3	0.2	–	–	–	–
CM0.4	1	0.3	0.4	–	–	–	–
CM0.6	1	0.3	0.6	–	–	–	–
LP0.25	1	0.25	1	–	–	–	–
LP0.35	1	0.35	1	–	–	–	–
LP0.45	1	0.45	1	–	–	–	–
UHPC-1	1	0.25	1	–	0.15	0.25	0.02
UHPC-2	1	0.25	–	1	0.15	0.25	0.02
UHPC-3	1	0.25	0.7	0.3	0.15	0.25	0.02

### Laser scabbling procedure

**Figure 1:**
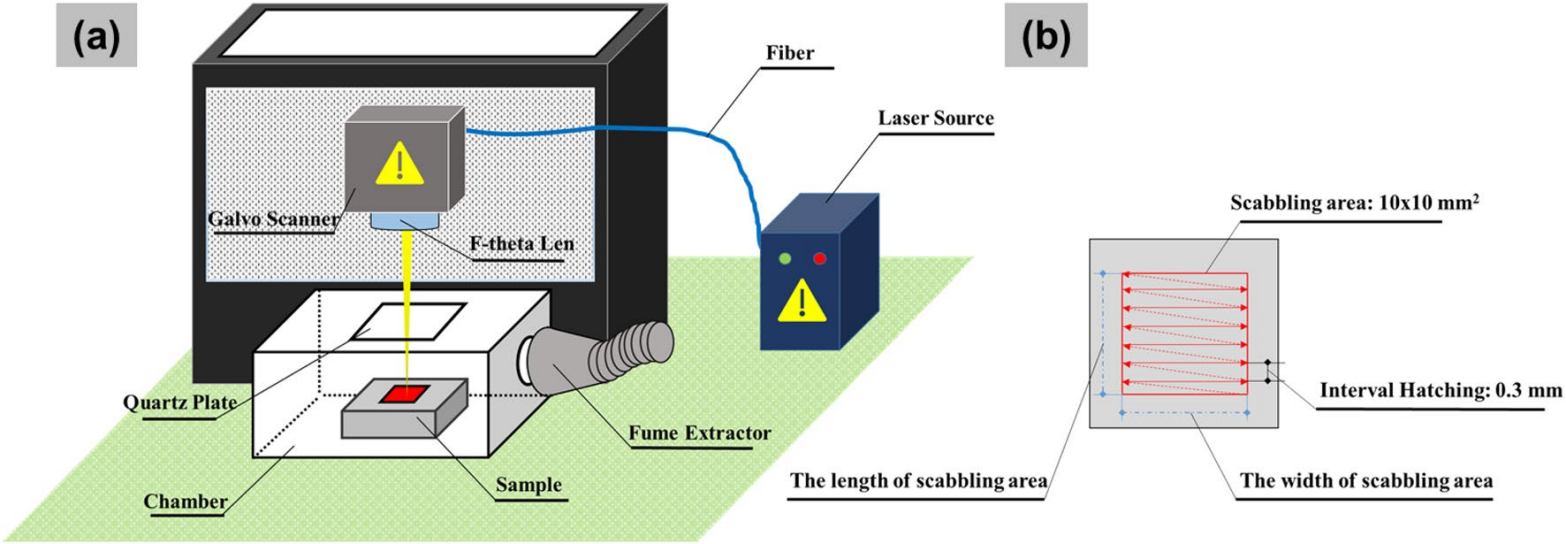
(**a**) Experimental setup, (**b**) Laser scabbling procedure.

The experiment was conducted using a 300 W Ytterbium pulsed fiber laser (IPG YLPN-2-20-500-300) operating at 1064 nm wavelength. The laser beam quality (M^2^) is 1.41. In this study, the output laser power was 250 W with a the focal spot size of 40 µm. The scabbling process proceeded with the pulsed laser mode. Details of the laser parameters applied for this study are listed in Table 2. The distance from F-theta Lens to the focal point on the irradiated sample surface was 180 mm. Each test sample was irradiated downward on an area of 10 mm (length) × 10 mm (width) for carrying out the scabbling process, as shown in Fig. 1. The scabbling path consisted of a square line and zigzag hatching lines with an interval hatching of 0.3 mm. The laser scabbling procedure was also shown in Fig. 1b**.** Furthermore, an experimental chamber was created to eliminate the negative effects of spatter during the testing process on the F-theta lens. Through an intake on the side of the chamber, the fume extractor was added and directly joined to the chamber to extract hot spatter and fumes, as illustrated in the Fig. 1a. Furthermore, a quartz plate with a thickness of 2.5 mm was installed on top of the chamber. UV–VIS spectrophotometer (SolidSpec-3700, Shimadzu) was used to measure the transmittance rate of quartz plates in the wavelength range of 200 to 2400 nm. The results showed that the transmittance rate of the quartz plates was 93.5% for the wavelength of 1064 nm. In fact, a laser meter measuring was carried out to measure the actual output power laser on the irradiated surface, and the value was recorded as 245.9 W in the case of a 250 W laser output.

**Table 2:** Laser parameters used in the experiment.

Laser parameters	
Output laser power (W)	250
Scanning speed (mm/s)	3;5;7
Pulsed duration (ns)	60
Pulse repetition rate (kHz)	1500
Laser spot diameter (µm)	40

### Analysis procedure

After the laser scabbling process, the surface observation was conducted using a confocal microscope Leica DVM6 (Leica Microsystems Ltd., Seoul, South Korea). And further, the selected samples were cut relatively in the center of the sample by using a mechanical cutter (Dewalt cutter, model-D28720). Following that, the samples were ground to provide a clear surface for observation by using a confocal microscope Leica DVM6. The observation zone was 17.46 mm (length) × 12.23 mm (width) × 11.35 mm (thickness). The specimen’s surface was then coated with a mixture of platinum (Pt) and Zirconium (Zr). A high-resolution scanning electron microscopy and EDX investigation were conducted inside the SEM machine (Mira CMH, TESCAN, Brno, Czech Republic). The beam intensity was set at 20.0 kW, with a working distance of 15.3 mm. Due to the inhomogeneity of concrete, at least three duplicates of each zone were measured.

## Results and discussions

### Morphological analysis

#### Surface observation analysis

**Figure 2:**
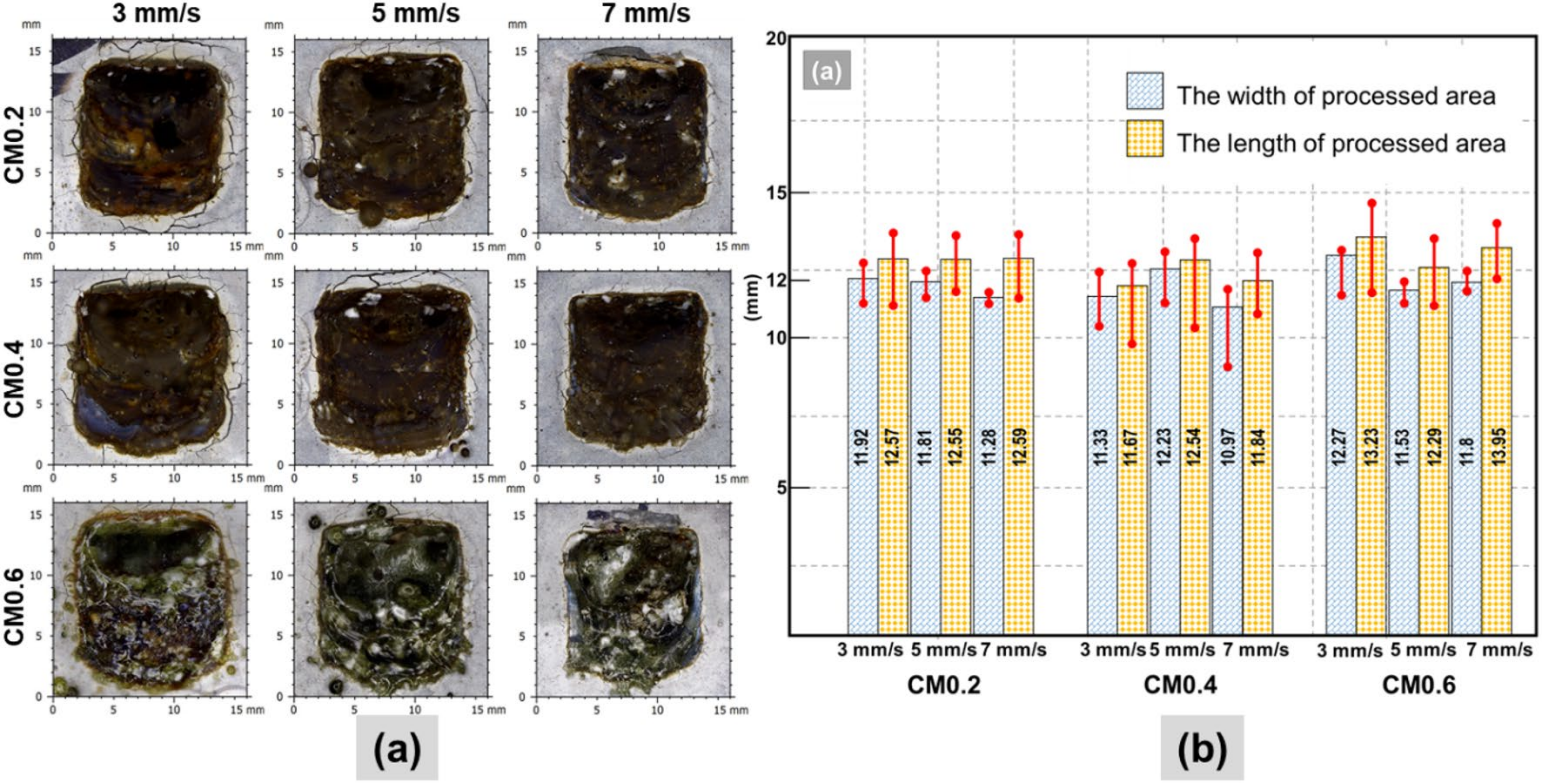
(**a**) Surface observation of CM series samples; (**b**) Dimension of CM series samples by using the confocal microscopy.

**Figure 3:**
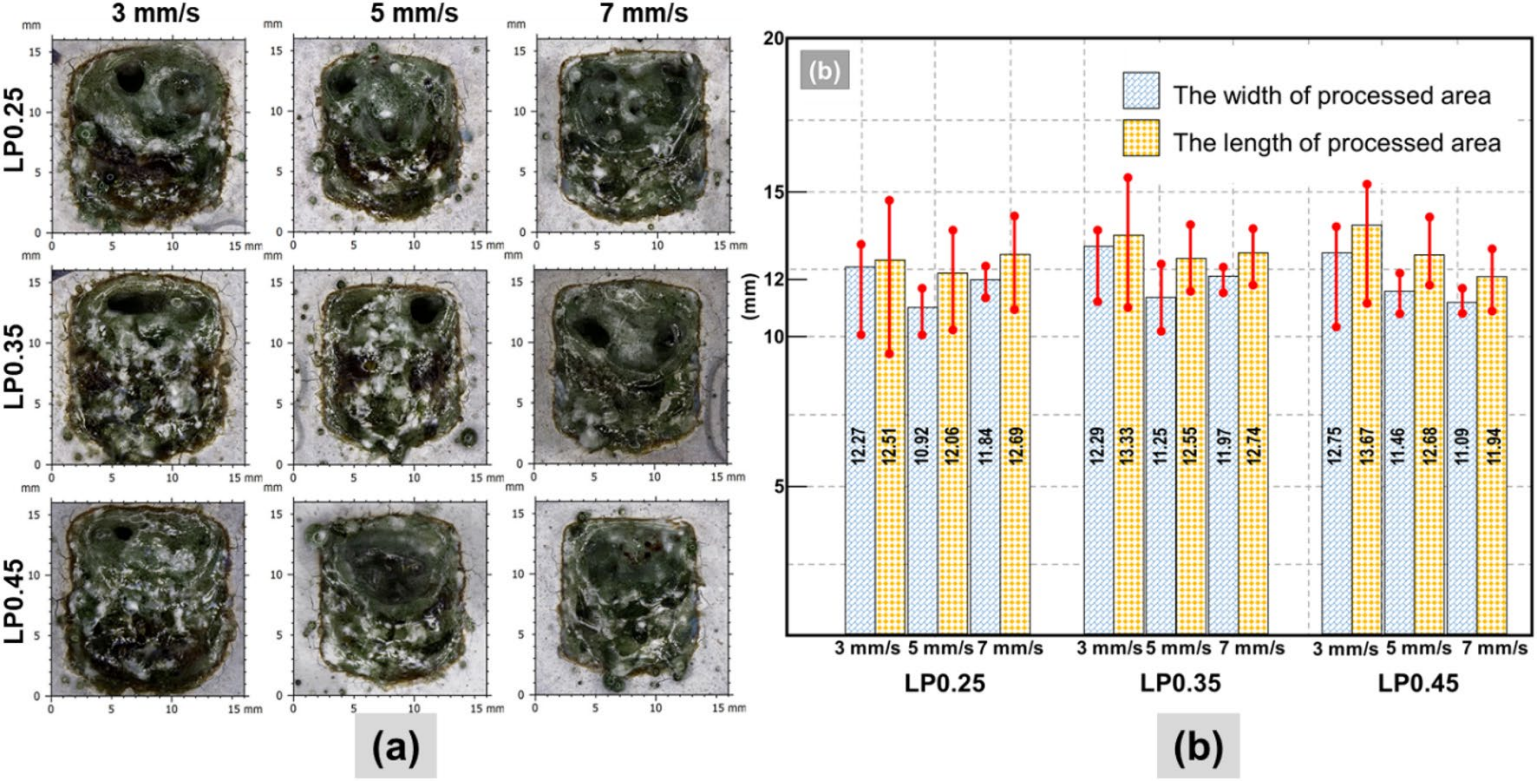
(**a**) Surface observation of LP series samples; (**b**) Dimension of LP series samples by using the confocal microscopy.

**Figure 4:**
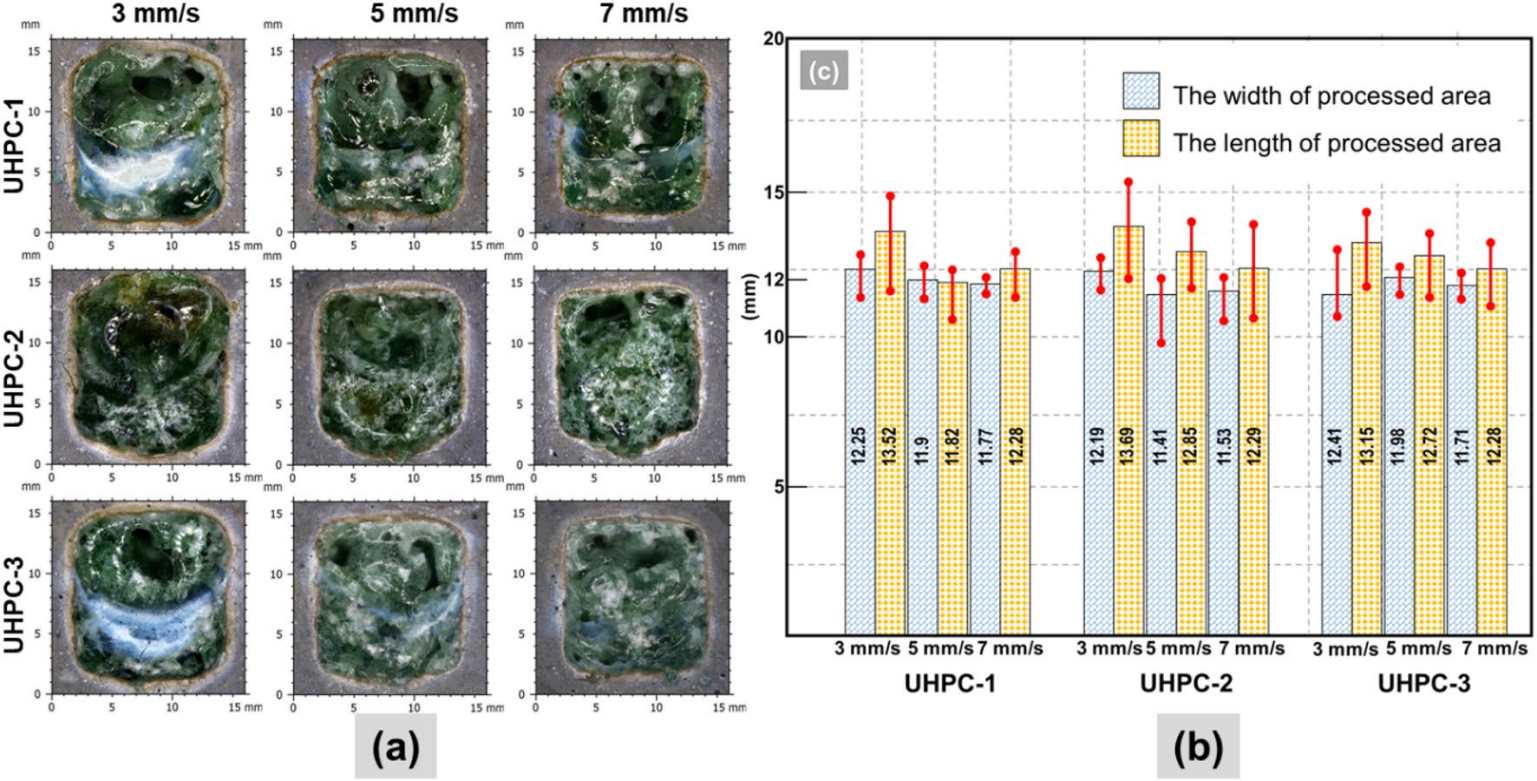
(**a**) Surface observation of UHPC series samples; (**b**) Dimension of UHPC series samples by using the confocal microscopy.

Two major findings of external appearance from the laser scabbling process were the generation of the glassy layer and the dimension variation of the scabbled area in all instances of CM, LP, and UHPC specimens, as shown in Figs. 2, 3, 4.

In the case of the CM series, the surface observations elucidated that increasing the proportion of silica sand in CM samples resulted in color change and an increased quantity of debris on the sample's surfaces, as depicted in Fig. 2a. Further, it was also noticed from Fig. 2a that the formation of cracks on the top surface was significantly reduced with increasing the proportion of silica sand. Besides, decreasing scanning speed resulted in more cracks and debris on the sample surface.

Surface observations for LP series are presented in Fig. 3a. It is quite evident that the LP series samples have a higher proportion of silica sand than the CM series samples, as seen in Table 1. The surface of the laser scabbled LP samples exhibited that lowering the laser scanning speed generates more debris and the formation of cracks. Unlike the CM series, the color changes were not observed in LP samples. Further, on the scabbled surface, the effect of water proportion was also ambiguous in LP series samples.

The surface appearance of the laser scabbled UHPC samples, as shown in Fig. 4a**,** exhibits a glassy layer inside the processed zone similar to the CM and LP series samples in Figs. 2a and 3a. In addition, the debris appearance dramatically decreased and barely be observed around the processed zone. One of the explanations for debris formation on the surface of the sample area was the influence of recoil pressure created during the laser process on cement-based material^17^. It is expected that the higher compressive strength and bonding properties of UHPC as compared to the cement mortar reduced the formation of debris on the surface of UHPC samples^30^. Compared to CM and LP series, for UHPC series samples, the influence of laser irradiation on cement-based materials showed significant color changes.

#### The dimensional variation of scabbled samples

After the scabbling process, a glassy layer was generated in the processed zone, as discussed above. The scabbling region was 10 × 10 mm^2^ as a set dimension. However, the differences in sample concrete composition and scanning speed led to the deviation of the desired dimensions inside the processed zone on the surface samples.

Figure 2b shows the dimension of the CM series, including the length and the width of the observation area. In most cases of the CM0.2 sample, the length and width of the processed area exhibited a minor variation among the other two CM samples. In the case of CM0.2, the average length of the processed area with laser scanning speeds of 3 mm/s, 5 mm/s, and 7 mm/s were 12.57 mm, 12.55 mm, and 12.59 mm, respectively. Besides, the average width of the processed scanning speeds of 3 mm/s, 5 mm/s, and 7 mm/s was 11.92 mm, 11.81 mm, and 12.28 mm, respectively. In most cases of scabbled samples, the width of the processed area was smaller than the length of the processed area. Especially, the dimension variation of the width was significantly smaller than the length in all results.

It should be noted that the label index of the LP series indicated the water proportion. Increasing the water proportion in the mixing sample resulted in the enlargement of the processed area at the lowest scanning speed of 3 mm/s, as seen in Fig. 3b. However, this tendency was not observable at the higher scanning speed such as 5 mm/s and 7 mm/s. The maximum mean length value of 13.67 mm was found in sample LP0.45 at 3 mm/s scanning speed, whereas the minimum mean length value was 11.94 mm, as measured in LP0.45 sample at 7 mm/s scanning speed. Compared with the CM series sample, the dimension variation of LP samples was higher. Since LP samples contained more water, the water evaporation phenomena during laser irradiation is expected to enhance the dimensional variations.

The dimension of the UHPC series samples is depicted in Fig. 4b. Overall, when compared to LP samples, the dimensional variation of UHPC samples was lower. With increasing laser scanning speed, the change in the mean width was insignificant, whereas the mean length was reduced significantly. Therefore, it can be observed that the length of the scabbled area was more strongly dependent on the laser scanning speed in all cases of UHPC samples. Furthermore, compared to the CM and LP series samples, the stability of the width of UHPC is more precise. Interestingly, the differences between the length and width of the processed area were subsequently confirmed to be low in the UHPC-1 sample at scanning speeds of 5 mm/s and 7 mm/s. The maximum length value was 13.69 mm at the UHPC-2 sample with a laser scanning speed of 3 mm/s. Similarly, the maximum length value was 13.15 mm at UHPC-3 with a laser scanning speed of 3 mm/s. The smallest dimension variation observed in UHPC-1 is mainly attributed to the fact that it contained type 1 silica sand as an aggregate component, as listed in Table 1**.**

#### Surface profile analysis of scabbled samples

**Figure 5:**
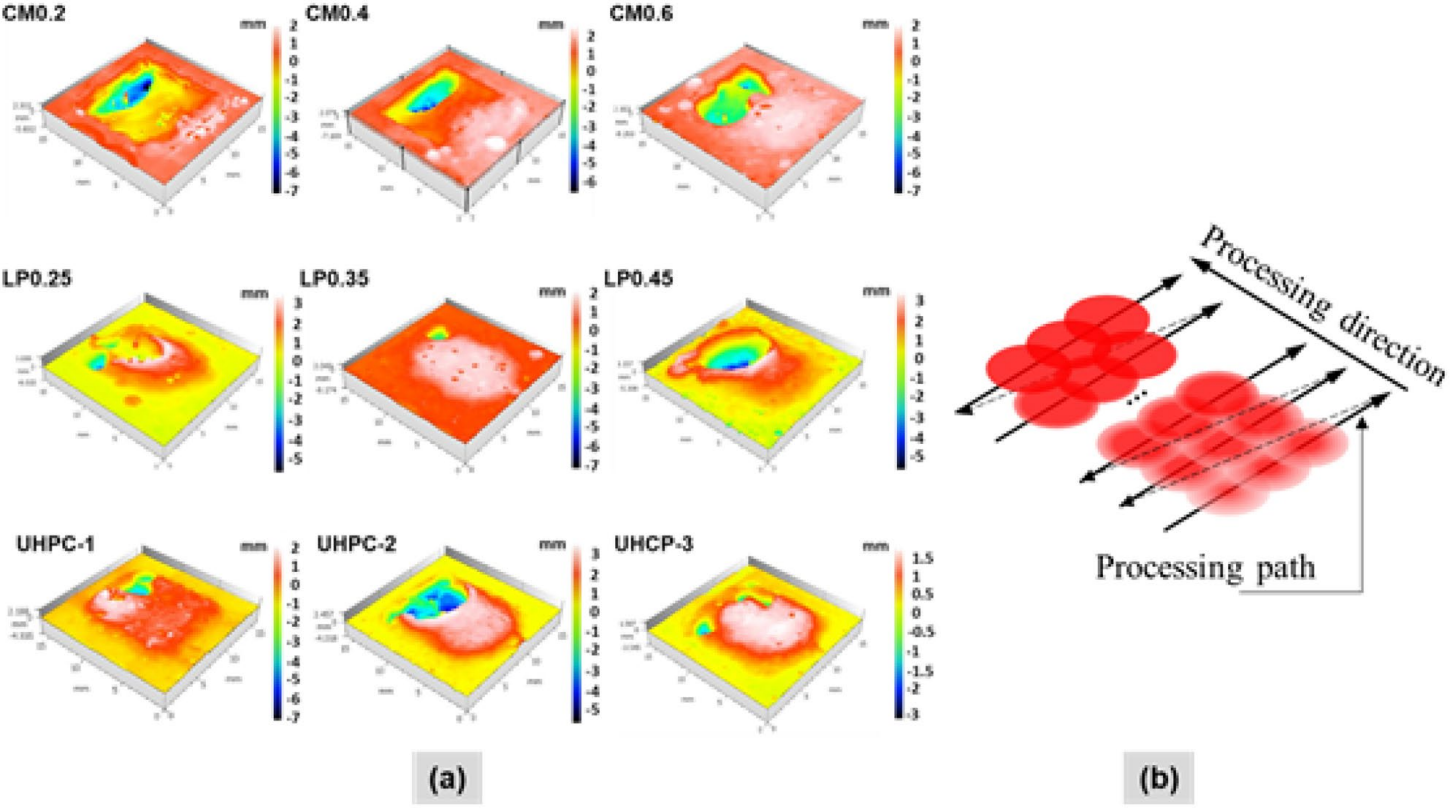
(**a**) 3D surface topography images of samples at scanning speed of 5 mm/s, (**b**) Schematic of laser pulsed heat accumulation.

Figure 5a,b show the surface profile of scabbled samples and laser processing path at the scanning speed of 5 mm/s. Besides, from the 3D topography surface, as shown in Fig. 5a, it was observed that the deeper scabbling depth was obtained at the end of the processing zone in most cases. In contrast, the scabbling depth was not achieved at the beginning of the processing for all CM, LP, and UHPC samples. At the initiation of the process, when the laser source irradiated the samples, the surface temperate quickly reached the glass transition temperature (~ 1300 °C)^10^, forming a hard glassy layer and accumulating some additional volume. At the end of the process, due to the heat accumulation as depicted in the Fig. 5b, the temperature surpassed the glassy temperature, resulting in the successful removal of the concrete from the samples^15,31^. It is expected that a higher amount of heat at the end of the processing zone is generated for lower laser scanning speed. Besides, the recoil pressure at the end process is higher as compared to the initial stage of process. The recoil pressure increases with the increasing of surface temperature^17^. Thus, the depression at the end of the scabbling process is also a result of the recoil pressure.

**Figure 6:**
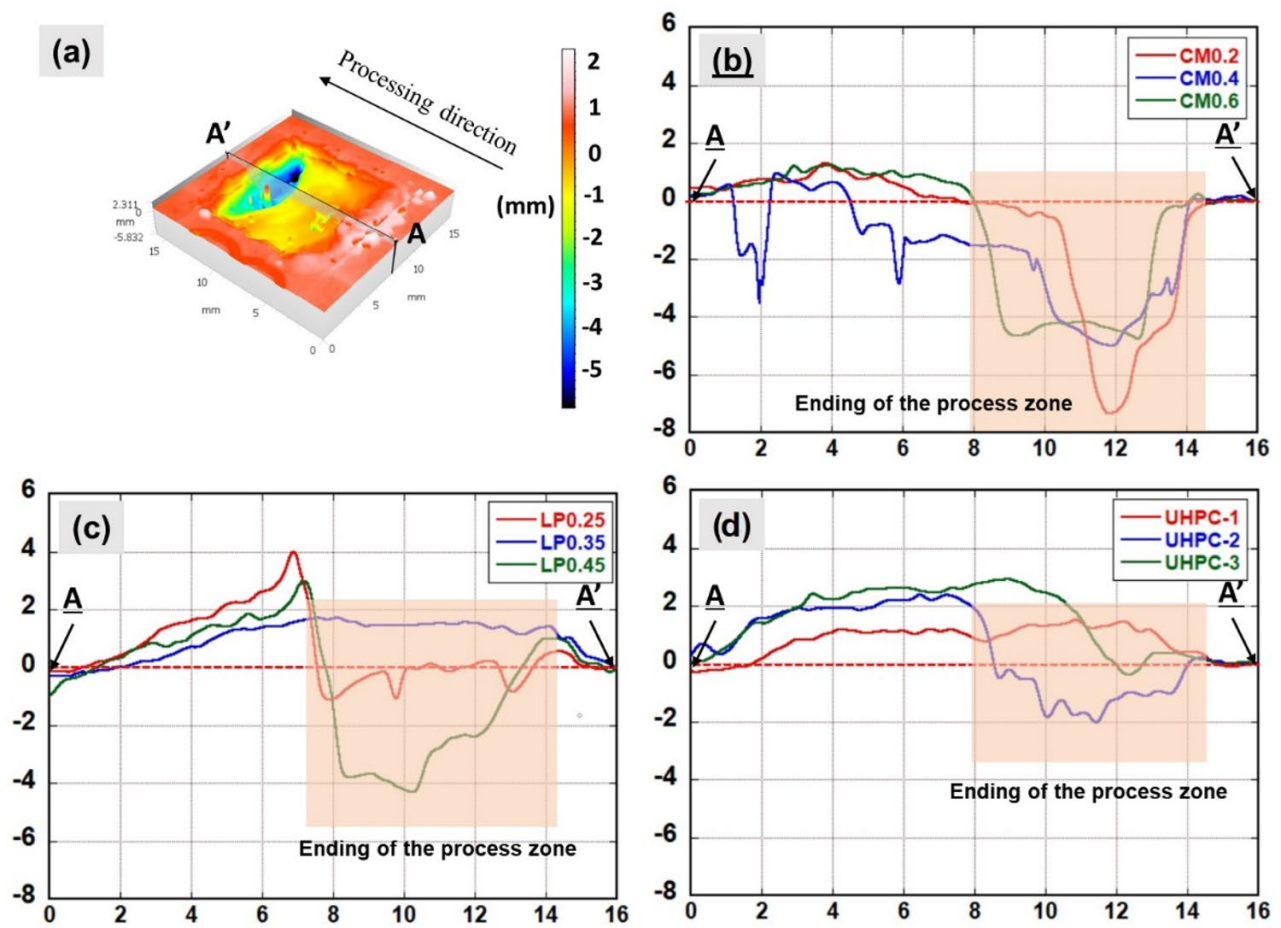
Cross-section profile of samples at 5 mm/s scanning speed: (**a**) 3D view of CM2; (**b**) A-A’ profile CM samples; (**c**) A-A’ profile LP samples; (**d**) A-A’ profile UHPC samples.

Figure 6a–d demonstrate the graphical representation of the depth profile for all the samples at the relative center of the scabbled zone. In Fig. 6a**,** along line A-A’, the scabbling depth was measured for the scanning speed of 5 mm/s. In the case of the CM series, the CM0.2 sample, which had the lowest silica sand proportion in mixing design, had the maximum scabbling depth, as depicted in Fig. 6b. The maximum in-depth value of the CM0.2 sample was 7.25 mm, whereas, in the CM0.4 and CM0.6 samples, it was 4.39 mm and 4.29 mm, respectively. This finding shows that the scabbling depth for CM samples depended on the proportion of silica sand. In case of CM0.4 samples the two sharp steep depth was formed at a distance of 2 mm and 6 mm. It is expected that during laser scabbling the gaseous bubble forming inside the glassy layer expanded and exploded to form holes on the surface^17^.

In the cases of LP samples, the scabbling depth decreased significantly compared to the CM series sample. The maximum scabbling depth value was measured at 4.04 mm at the highest water proportion sample of LP0.45, as displayed in Fig. 6c. As compared with the CM and LP series samples, the scabbling depth of UHPC samples significantly decreased, as exhibited in Fig. 6d. The maximum scabbling depth was measured at 2.21 mm for the UHPC-2 sample, mixed with type 2 silica sand with the smallest mean dimension of 0.25 mm. Thus, it can be implied that the smaller aggregate size can also affect the scabbling depth.

**Figure 7:**
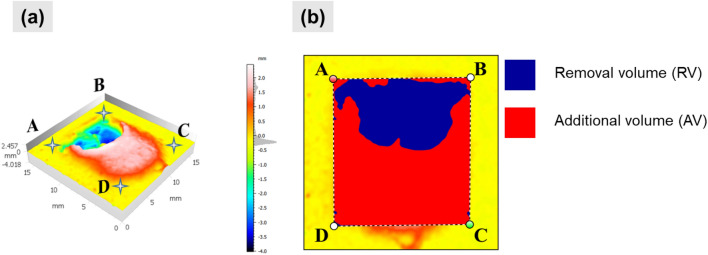
(**a**) UHPC-2 at scanning speed of 5 mm/s 3D Topography surface images, (**b**) RV and AV definition.

The 3D topography study revealed additional volume (AV) and removal volume (RV) formation post scabbling process. Thus, an in-depth study was carried out for all samples at three different laser scanning speeds. An additional volume was determined by the volume of the glassy layer produced above the original reference surface of the sample. On the other hand, the removal volume was defined by the void formed after scabbling on the material under the original reference surface of the sample, as illustrated in Figs. 7a,b.

**Figure 8:**
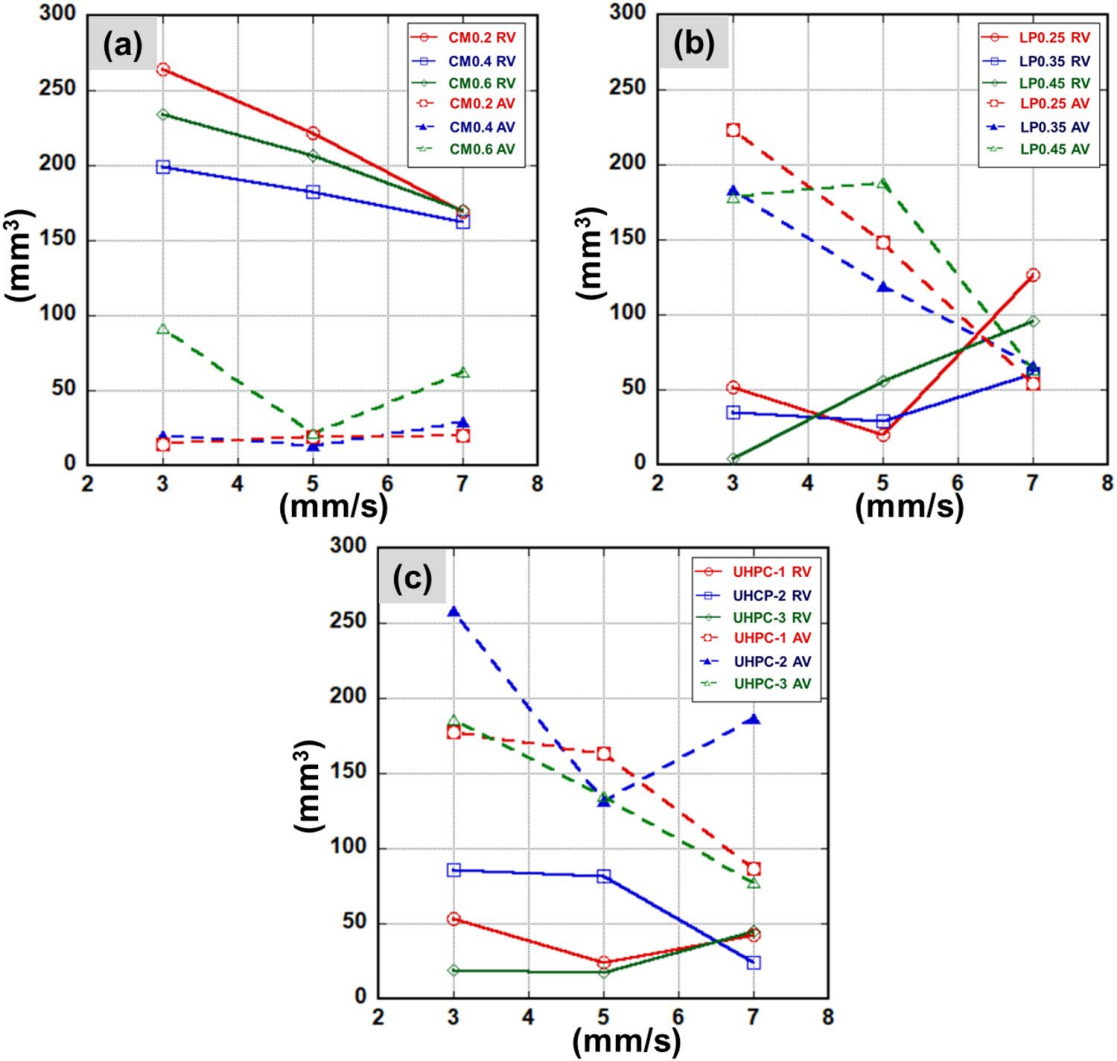
Removal volume (RV) and Additional volume (AV) of laser scabbling samples: (**a**) CM series, (**b**) LP series, (**c**) UHPC series.

Figure 8a–c describes the volume changes depending on the scanning speeds and material types. Overall, the AV was lower than the RV in the CM samples, as shown in Fig. 8a. The maximum RV was measured at the CM0.2 sample of 264.3 mm^3^ at the laser scanning speed of 3 mm/s. The higher silica sand proportion in CM samples and faster laser scanning speed led to the reduction of RV. In addition, the formation of AV in the CM series sample was insignificant except for the CM0.6 sample.

In contrast, AV formed significantly in cases of LP and UHPC samples, as shown in Figs. 8b,c**.** In the cases of LP samples, increasing laser scanning speed led to the significant formation of AV and reduction of RV. The maximum RV value was obtained for LP0.25 of 223.5 mm^3^ when the laser scanning was 3 mm/s. However, at the laser scanning speed of 5 mm/s, the RV of LP0.45 was 188.1 mm^3^ and greater than LP0.25 and LP0.35, which had RVs of 148.8 mm^3^ and 118.9 mm^3^, respectively. Further, at the laser scanning speed of 7 mm/s, it was noticed that the difference of RV for LP samples is insignificant. At the laser scanning speed of 7 mm/s, the AV became significantly higher than RV in LP samples.

In UHPC samples, the AV was entirely higher than RV, as shown in Fig. 8c. Similar to the scabbling depth observation, the maximum RV of UHPC was measured for the UHPC-2 samples of 86.13 mm^3^ when the laser scanning speed was 3 mm/s. Meanwhile, the RV of UHPC-1 and UHPC-3 was not much different. Thus, it can be concluded that the aggregate size played an essential role in the laser scabbling, as the UHPC-1 and UHPC-3 samples were fabricated with aggregate having a larger size (1.45 mm) than the UHPC-2 sample (0.25 mm). Further, it is also true that the highest amount of silicate proportion in the UHPC sample fabrication led to the significant formation of the glassy layer, which also restricted the penetration of the laser beam.

The above results show that AV is greater than RV, mostly for LP and UHPC samples. The RV and AV depend mostly on the laser scabbling parameters and the composition of the cement materials. The additional volume, as discussed above, refers to the glassy layer formation whose nature is entirely different from the cement-based materials. Thus, it can be interpreted that laser interaction with the concrete can remove the material and change its characteristics too. It is expected that the formed glassy layer can trap the radioactive surface of the concrete wall during the decommissioning of the nuclear power plants.

### Microstructural analysis

#### Formation of different zones after laser scabbling

**Figure 9:**
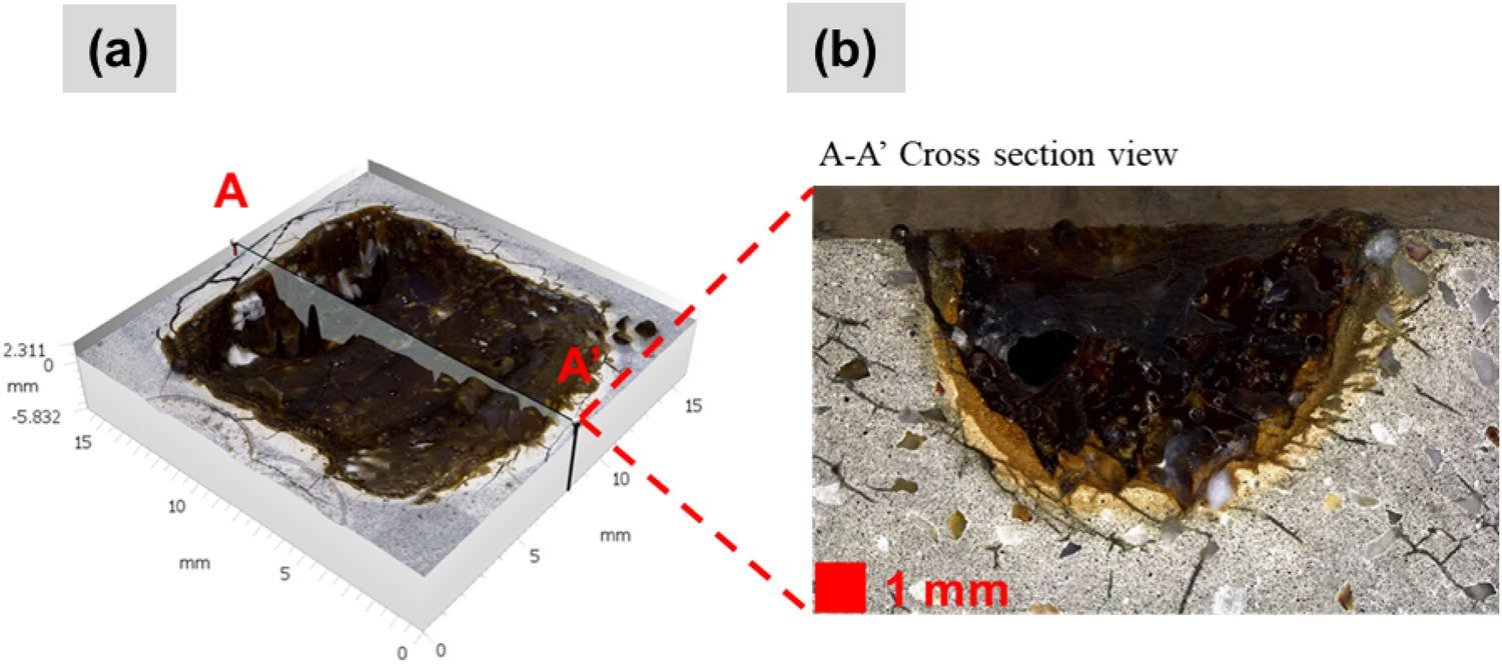
(**a**) Cross-section preparation, (**b**) Cross-sectional view of CM0.4 sample at 5 mm/s scanning speed.

After the laser scabbling process, one sample from each of the three types (CM, LP, and UHPC) was chosen for the microstructural analysis. To study the effect of water and silica contents, LP0.45 was selected as the material with the highest water content, whereas CM0.4, with low silica sand content, and UHPC-2 the highest. Figure 9a,b show the preparation of the cross-section and the characteristics of the CM0.4 sample**.**

**Figure 10:**
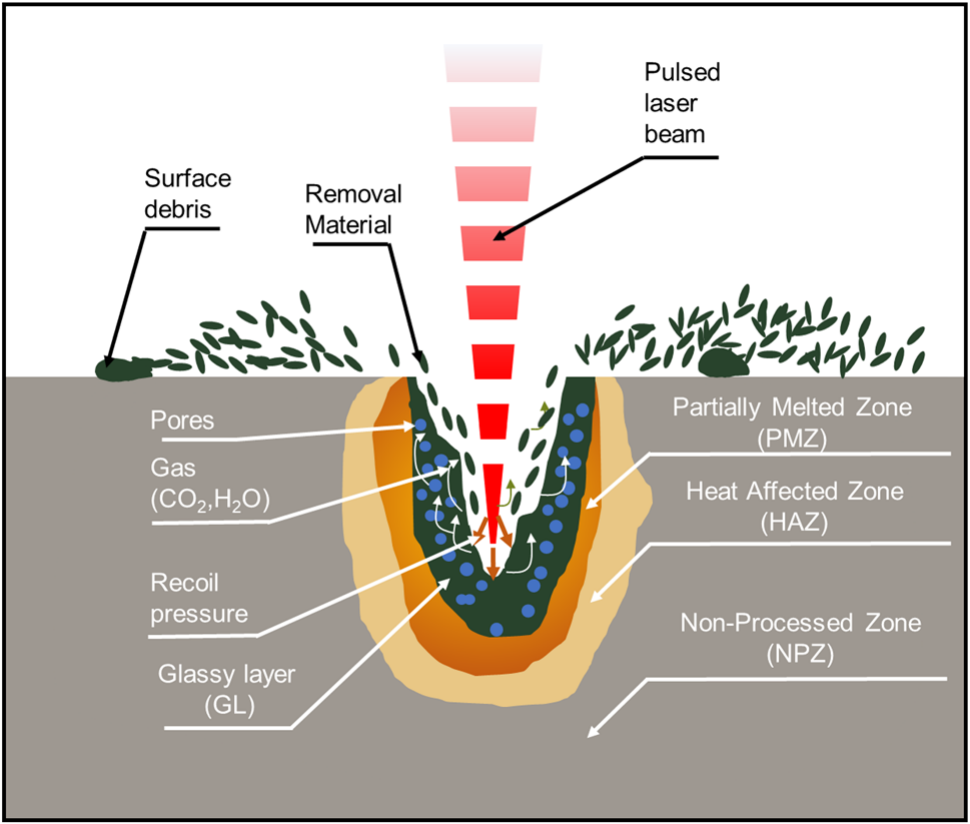
Illustrator images of 4 main zones in cross-section view.

According to the results observed from the cross-sectional view, four main zones of samples were spotted such as a glassy layer, a partially melted zone (PMZ), a heat-affected zone (HAZ), and a non-processed zone, as illustrated in Fig. 10.

When a laser pulse interacted on the cement-based material surface, the sample was rapidly heated by the absorption of the energy. Besides, the chemical and physical changes of elements in cement-based materials under the influence of laser-assisted heating resulted in the generation of glassy layers mainly consisting of SiO_2_. Several physical and chemical reactions are caused by the dehydration and decomposition process of cement-based materials during laser processing. The dehydration process of heated cement pastes and changes in the phase of aggregate elements resulted in the color changes of heated cement-based material^29,32^. In addition, the color changes of the processed zone that generated a glassy layer are visually observed compared to the other areas^33^. Wignarajah et al.^34^ reported that the presence of metallic oxides in the cement samples produced color on the surface layer affected by the laser. Although the glassy layers contain mostly SiO_2_, the color of the glassy layer also appears due to the laser's interaction with metallic oxides present in the material composition, as listed in Table 3.

**Table 3:** Examples of colored glassy layers produced on the surface of zeolite mortar by laser irradiation^32^.

Type of oxide	Color produced
Cr_2_O_3_	Light to dark green
CoO	Light to dark blue
MnO_2_	Brown
CuO_2,_ Cu_2_O	Brown, black, brick red
FeO, FeO_2_, Fe_3_O_4_	Grey to black

**Figure 11:**
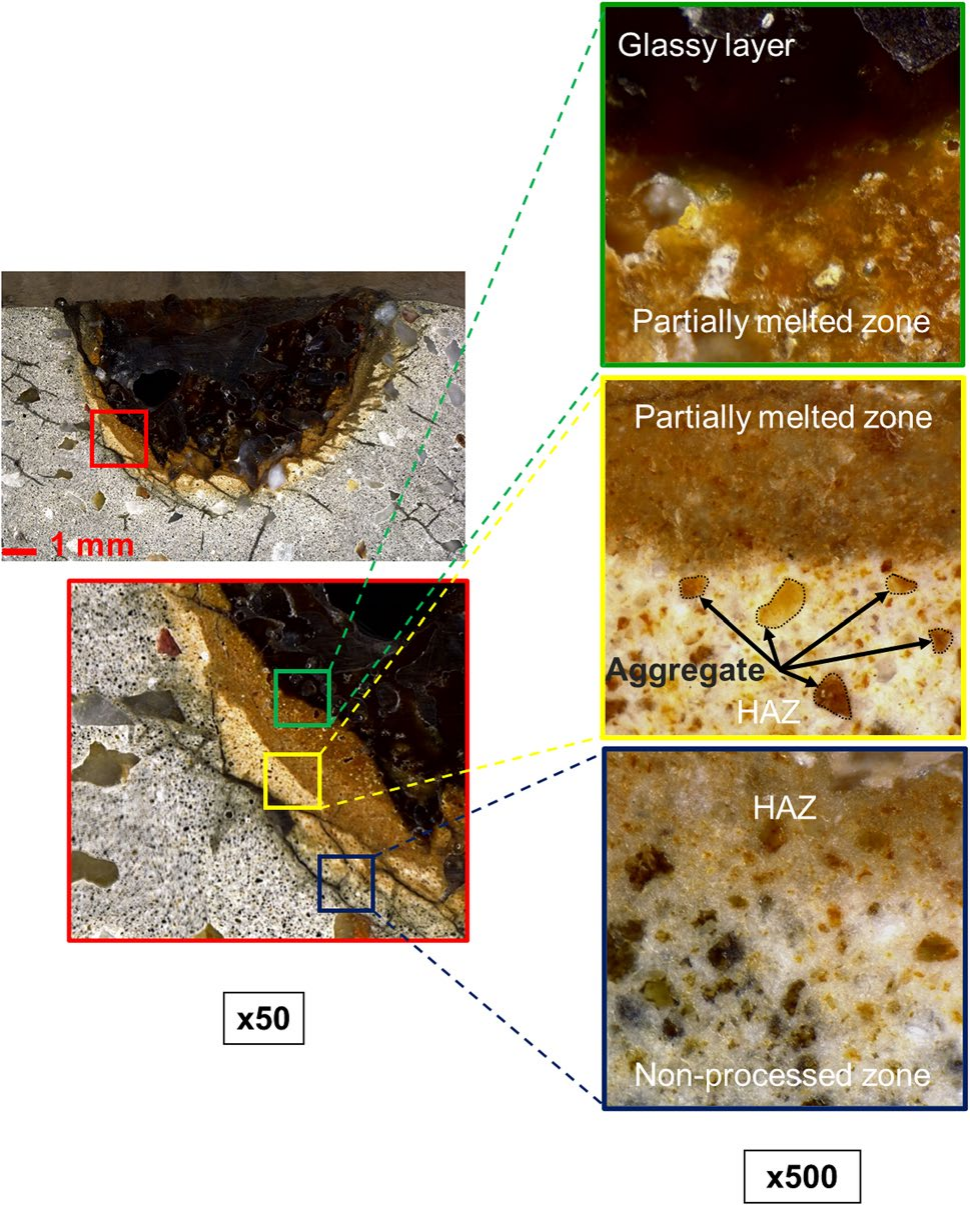
The appearance of 4 zones of sample CM0.4 at 5 mm/s scanning speed.

The partially melted zone was found between the glassy layer zone and HAZ. Figure 11 presents an example of the four main zones of the sample after the laser scabbling process. Due to the direct interaction of laser irradiation on the surface sample, the temperature at the processed zone was the highest in the laser scabbling process. It gradually decreased in other zones as the heat was transferred to surrounding zones. Different types of phase changes of concrete components such as cement and aggregate were observed in the glassy layer zones, PMZ, and HAZ due to the difference in the thermal gradient. Furthermore, it was considered that the color change in the glassy layer, PMZ, and HAZ was a distributed consequence of the laser irradiation temperatures.

Inside the partially melted zone, it is expected that the temperature mostly varied between the 600–1000 °C range. This further initiated the formation of the β-C_2_S, change in the second phase of C–S–H, and dolomite decomposition. Besides, Portlandite decomposition and quart phase change β-α in aggregate and sand resulted in the formation of PMZ^35–37^.

**Figure 12:**
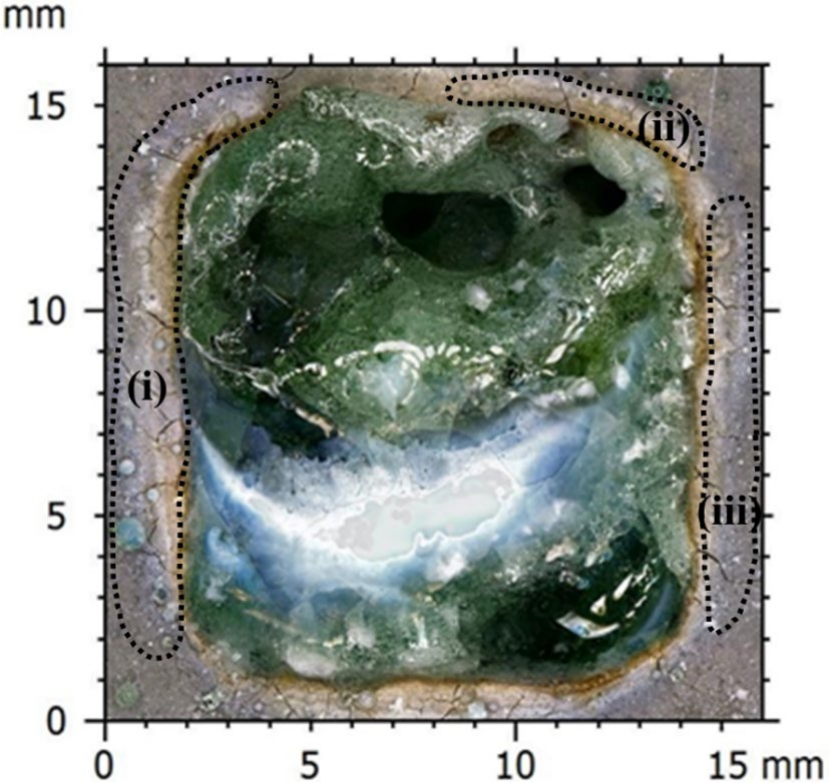
(i–iii) Top surface HAZ, cracks appearance.

HAZ was indirectly affected by the heat propagation of laser irradiation on the processed zone. The color of the HAZ changed and appeared on the top surface with a whitish-grey color, as shown in Fig. 12**.** However, in the cross-section surface, a part of HAZ was shown in bright yellow color, and the aggregates were not melted and maintained their presence in the HAZ, as shown in Fig. 11. The cracks spotted inside the HAZ are mainly generated due to the loss of bound water caused by C–S–H decomposition and decarbonization of calcium carbonate^37,38^.

#### The pore formation and microstructure of different zones

**Figure 13:**
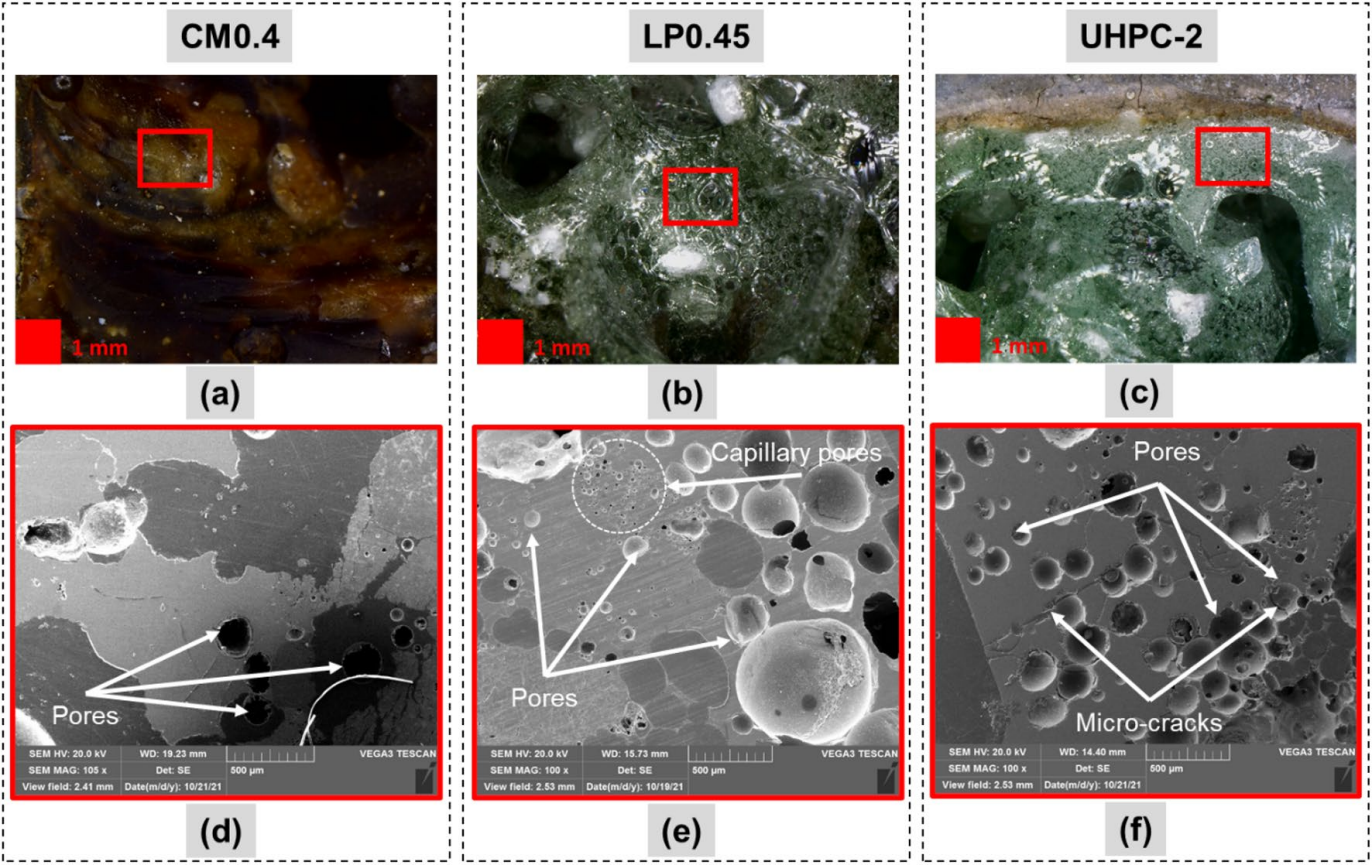
The bubble appearance in the top (**a**–**c**) and pore structure (**d**–**f**) in the cross-section of the glassy layer at the laser scanning speed of 5 mm/s.

Figure 13a–c show the glassy layer with the clear pores in cases of LP0.45 and UHPC-2 samples. However, the pore observation was unclear in the cases of CM0.4. Scanning electron microscopy (SEM) was also used with a magnification of 100 × to identify the appearance of air bubbles inside the glassy layer, as exhibited in Fig. 13d–f. The pores formed more densely in the UHPC-2 sample with a diameter of 100–300 µm, as shown in Fig. 13f. These pores formed due to gases like H_2_O vapor and CO_2_ entrapment during the laser scabbling process. Approximately 6% of unbound water is contained in cement-based material^10^. Thus, the water molecules present in the cement-based material evaporated under high temperatures heated by laser irradiation. Moreover, CO_2_ was also a byproduct of the dehydrating process under high temperatures of primary components like C–S–H, Ca(OH)_2_, and CaCO_3_ inside the cement paste. Furthermore, a higher quantity of pores was generated for LP0.45 and UHPC-2 than in the CM0.4 sample, as revealed in Fig. 13d–f. Due to the high evaporation temperature of silica sand, the higher silica sand proportion in the LP0.45 and UHPC-2 sample generated a glassy layer maintaining the high viscosity of the molten pool, which minimized the releasing of the gases and generated a higher amount of pores as discussed above^17^.

**Figure 14:**
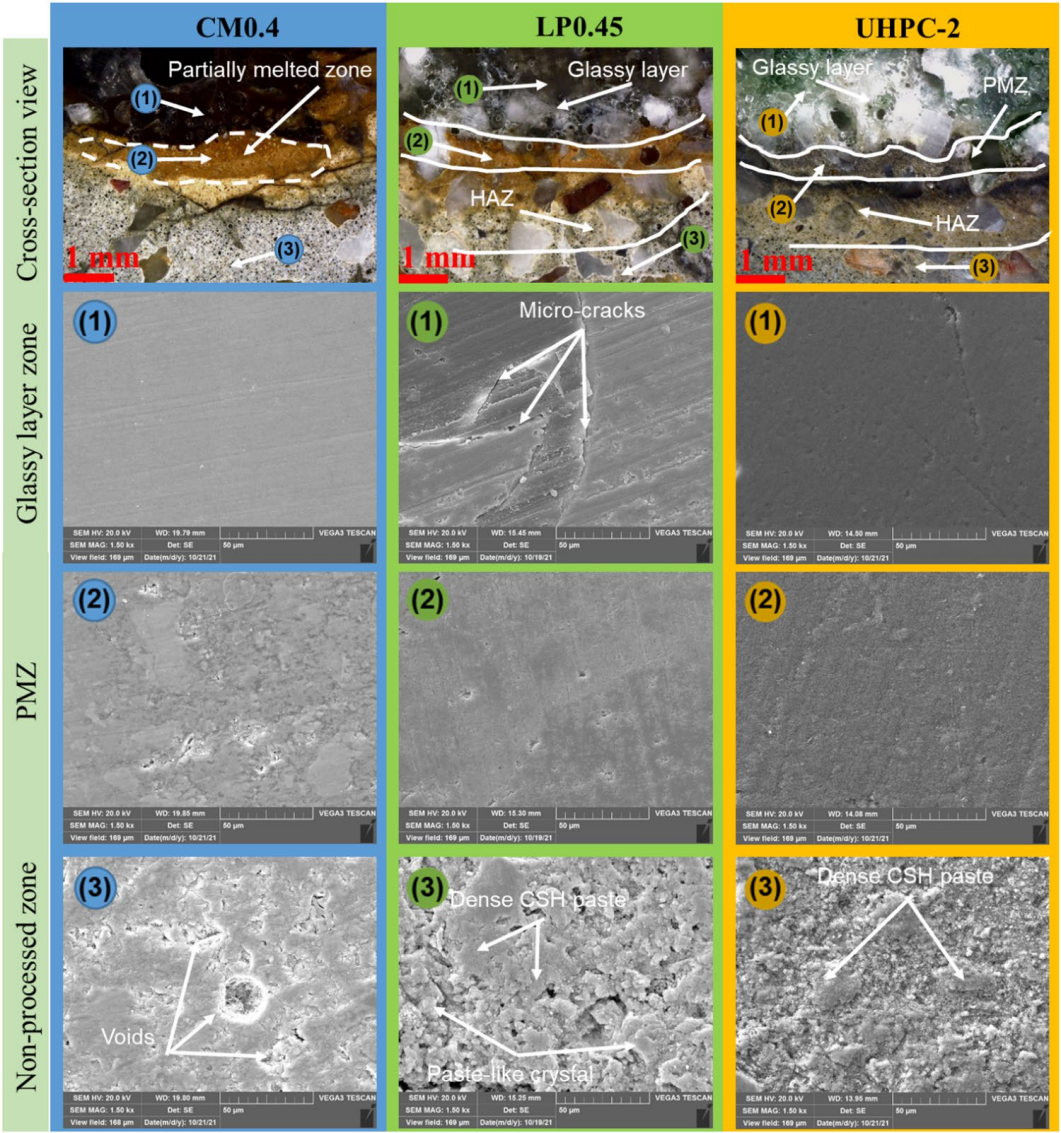
Microstructure of Glassy layer, PMZ, and non-processed zone on cross-section views of CM0.4, LP0.45, and UHPC-2 samples.

In-detailed microstructural changes were studied for similar samples using a 1500 × magnification. Cement paste, calcium hydroxide (CH), calcium–silicate–hydrate (C–S–H) gel, and crystal hydration were observed as primary components on the surface of the non-processed zone. The portlandite content rapidly decreased during the heating process by Ca(OH)_2_ → CaO + H_2_O↑^39^. Thus, dehydrated cement and decomposed product of C–S–H are expected to form inside the PMZ. Inside the processed zone under laser irradiation, cement paste decomposed completely, and silica sand was wholly melted, resulting in a glassy layer zone with a dense structure containing a mixture of SiO_2_ and CaO. Besides, the structure of PMZ and the glassy layer zone indicated a denser structure when compared to the non-processed zone, as shown in Fig. 14. It is expected that the denser structure in the PMZ was generated due to the decomposition of C–S–H and formation of coarse β-C_2_S and C_3_S reducing the pore diameter inside the cement material^40^.

### EDX analysis

**Figure 15:**
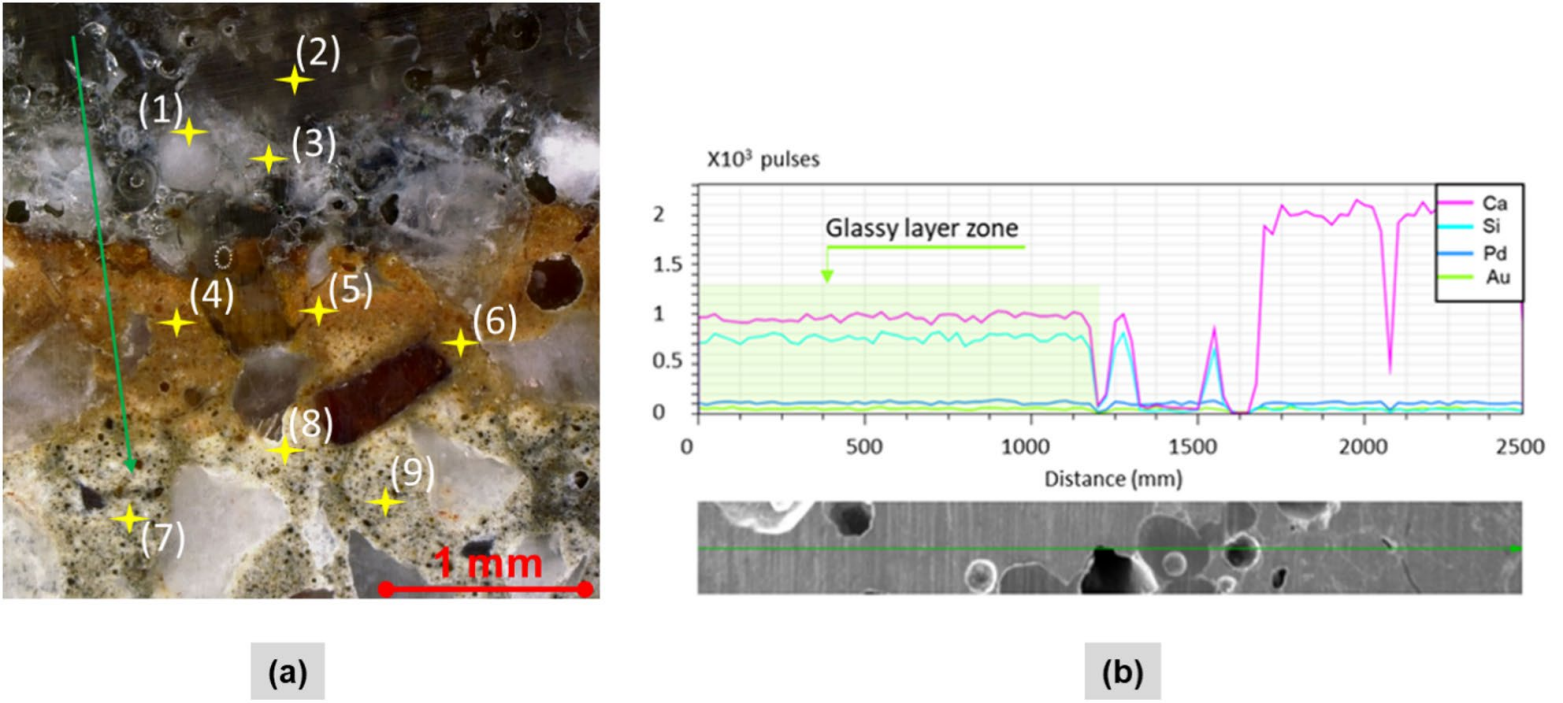
(**a**) Chosen points for chemical analysis in the cross-section, (**b**) Identification of the glassy layer based on the EDX line scan.

Sample LP0.45 with a scanning speed of 5 mm/s was analyzed in detail. Several points were chosen, and the chemical changes of each point are provided in Table 4. It is essential to highlight that the chemical composition of the cross-section surface was determined by experimenting with 9 points. Points number 1, 2, and 3 were located in the glassy layer, whereas points 4, 5, and 6 were located in PMZ. Both HAZ and non-processed zones were pointed as 7, 8, and 9. In addition, most of the selected points avoided selecting points on the silica sand particle, as shown in Fig. 15a.

**Table 4:** Composition (wt %) by EDX analysis of LP0.45 with a scanning speed of 5 mm/s. Measured points are indicated in Fig. 15.

Point #	Calcium	Silicon	Iron	Oxygen	Aluminum	Kalium	Magnesium	Phosphorus
1	50.49	22.5	9.81	14.7	0.35	0.68	1.26	0
2	43.94	18.13	10.89	25.3	1.25	0.49	0	0
3	45.36	20.98	11.07	17.6	3.26	0.25	0	1.48
4	60.55	15.6	9.29	12.3	0.65	1.61	0	0
5	69.23	12.35	1.66	14.03	0.14	1.24	1.35	0
6	76.05	6.65	9.71	5.68	1.26	0.65	0	0
7	56.62	7.12	8.06	24.6	0.36	2.36	0	0.68
8	63.34	8.12	6.60	18.95	0.64	1.53	0.68	0.14
9	68.7	6.24	5.65	14.8	0.98	1.11	1.65	0.87

Observing chemical changes in zones of cross-section revealed a large amount of metals such as iron and aluminum with an increase in the percentage of silicon and a decrease in the calcium inside the glassy layer. The partially melted zone exhibited lower silicon but higher calcium content than the glassy layer. Oxygen presented in each formed zone. Specifically, Fe and Al and other metallic elements found in the concrete influence the formation of a different color when exposed to high temperatures found in cement-based material^34,41^. Thus, the colored surface forming inside the glassy layer is due to the presence of various metallic oxides. The cement pastes and silica sand melt when the surface temperature reaches 1300–1600 °C^10^. The formation of SiO2 is mainly attributed to the significant increase in silicon content inside the glassy layer during laser irradiation of samples that contained a high amount of silica sand. The evaporation temperature of calcium explains calcium depletion in weight percentage, which is around 1400 °C, and it is significantly lower than the evaporation temperature of silicon (3170 °C).

The use of the EDX line is shown in Figs. 15a,b. The line scan was chosen to avoid scanning through silica sand particles. The result is shown in Fig. 15b also revealed the increased presence of silicon in the glassy layer. A decrease in silicon and an increased amount of calcium can be seen outside the glassy layer since the main component of the cement paste is calcium mainly. The glassy layer can be easily identified by using the EDX line scan technique as it exhibits compositional differences with the concrete material. At the same time, it is not easy to locate other zones such as PMZ and HAZ as the difference in silicon and calcium is not significant.

## Conclusions

This study successfully identified the effect of laser on nine different types of cement-based material, including cement mortar and ultra-high-performance concrete (UHPC). The influence of variations in silica sand, water proportion, and mineral components in the laser scabbling process was discovered experimentally with confocal microscopy, scanning electron microscopy, and 3D surface topography. The cumulative results obtained from the experimental observations drew the following conclusive remarks.

- The increase in silica sand proportion in cement mortar revealed the rise of debris and decreasing cracks formation along the processed zone.
- After laser scabbling, the color of the glassy layer was not affected by the proportion of water in cement-based material.
- The difference in scabbling depth at the beginning and end of the laser processing zone resulted from heat accumulation during the laser processing period.
- The removal volume decreased in the CM samples by increasing the laser scanning speed. Whereas, the removal volume in LP and UHPC was significantly reduced compared to CM samples.
- In higher silica sand proportion in cement-based materials such as LP and UHPC samples resulted in a significant increase in additional volume and a reduction in removal volume. The formation of additional volume was lower in CM samples than in LP and UHPC samples. The processed zone for these two samples generated a viscous glassy layer entrapping gases like CO_2_ and vapors of H_2_O, resulting in higher pores inside the glassy layer.
- The four different zones, namely the glassy layer, PMZ, HAZ, and non-processed or unaffected, exhibited variation in color appearances, mostly due to different thermal gradients enforcing the various phase changes within. Interestingly, the HAZ on the surface revealed a whitish-grey appearance, whereas a yellowish color was observed inside the cross-section.
- Inside the glassy layer, a significant rise in silicon and metallic elements like iron and aluminum occurred, whereas calcium percentage decreased significantly. The presence of oxygen was also detected. Combining these findings mostly justifies the presence of SiO_2_ complete melting of calcium-enriched cement paste and coloring of the metallic oxides.

## Data Availability

The datasets used and/or analyzed during the current study are available from the corresponding author on reasonable request.
